# Educational attainment, brain cortical structure, and sarcopenia: a Mendelian randomization study

**DOI:** 10.3389/fpubh.2024.1415343

**Published:** 2024-10-23

**Authors:** Yunqing Zhang, Ruideng Wang, Zhengyang Chen, Fang Zhou, Shilong Su

**Affiliations:** ^1^Department of Orthopedics, The First Hospital of Changsha, Changsha, China; ^2^Department of Orthopedics, Peking University Third Hospital, Beijing, China; ^3^Engineering Research Center of Bone and Joint Precision Medicine, Peking University Third Hospital, Beijing, China

**Keywords:** educational attainment, brain cortical structure, sarcopenia, Mendelian randomization, causal effect

## Abstract

**Background:**

Previous observational studies have suggested associations between high-level educational attainment (EA) and a lower risk of sarcopenia. However, the causality inferred from those studies was subjected to residual confounding and reverse causation. The protective effect of EA on sarcopenia may be mediated via changes in brain cortical structure. The aim of this study was to use a two-step Mendelian randomization (MR) analysis to illustrate the causal relationship between EA, brain cortical structure, and sarcopenia.

**Methods:**

Instrumental variables at the genome-wide significance level were obtained from publicly available datasets, and inverse variance weighted as the primary method was used for MR analysis. We perform several sensitivity analyses, including Cochran Q test, MR-Egger intercept test, leave-one-out analyses, and MR Pleiotropy Residual Sum and Outlier to evaluate the reliability of the results.

**Results:**

EA was causally associated with increased appendicular lean mass (*β* = 0.25, 95% confidence interval (CI): 0.19 to 0.31, *p* = 2.25 × 10^−15^), hand grip strength (left: *β* = 0.042, 95% CI: 0.013 to 0.071, *p* = 4.77 × 10^−3^ and right: *β* = 0.050, 95% CI: 0.022 to 0.079, *p* = 5.17 × 10^−4^), and usual walking pace (β = 0.20, 95% CI: 0.18 to 0.22, *p* = 6.16 × 10^−83^). In addition, EA was associated with increased brain cortical surface area (*β* = 4082.36, 95% CI: 2513.35 to 5681.38, *p* = 3.40 × 10^−7^) and cortical thickness (TH) (*β* = 0.014, 95% CI: 0.0045 to 0.023, *p* = 3.45 × 10^−3^). Regarding the causal effect of EA on usual walking pace, the mediatory effect of TH was 0.0069 and the proportion of mediation by TH was 3.43%.

**Conclusion:**

The study will have revealed the protective causal effect of EA on sarcopenia, which provides a reference for the prevention of sarcopenia at the public health level. We also will have found EA could affect the brain cortical structure, and the brain cortical structure could mediate the protective effect of EA against sarcopenia risk.

## Introduction

Sarcopenia is an age-related condition featured by a progressive and generalized skeletal muscle disorder that involves the accelerated loss of muscle mass and function (1). In addition to usually affecting the older adult, it can also occur in middle age (1) and is prevalent in patients with certain diseases, such as cancer (2) and metabolic disorders (3). Sarcopenia is associated with an increased risk of falls, fractures, functional decline, and mortality (4). These symptoms and risks seriously reduce the quality of life of patients and place a greater burden on families and society (4). The prevalence of sarcopenia in the older adult is about 5–12.9% (5, 6). With the aging of the population, sarcopenia may impose an increasing burden on medical and health institutions in the future. Sarcopenia has gradually become a public health problem (1). However, the etiology and risk factors of sarcopenia are still not fully described (1, 7). It is therefore necessary to identify potential causal risk factors that will help guide prevention efforts.

Several observational studies have reported that a high level of educational attainment (EA) was associated with a lower risk of sarcopenia (8–12). However, these observational studies were prone to be influenced by confounding factors and reverse causality (13). Although randomized controlled trials are the gold standard for establishing causality, so far, there have been no randomized controlled trials to investigate the effects of EA on sarcopenia, which may be due to practical or ethical issues (7). In fact, clarifying the causal relationship between EA and sarcopenia is of broad significance for understanding the etiology and prevention of sarcopenia. Faced with sarcopenia, which is gradually becoming a public health problem, it is not enough to just treat patients. It is necessary to study its epidemiology and reform public health policies to prevent more cases from occurring. Moreover, observational neuroimaging studies in the older adult have shown that education was related to magnetic resonance imaging (MRI) measurements of brain structure. For example, higher levels of EA were associated with increased volume of gray matter in the whole brain and regions (14, 15), increased cortical thickness (16), and increased cortical surface area (17). At the same time, more and more studies have confirmed the existence of muscle-brain cross-talk through a variety of cytokines and neuromuscular junctions (18, 19). Given the existence of these associations, we have reason to assume that if the causal effect between EA and sarcopenia is confirmed, then changes in brain cortical structure may mediate the protective effect of EA against sarcopenia risk. However, interpreting these observed associations means that the causal relationship between EA, brain cortical structure and sarcopenia needs to overcome the interference of confounding factors and reverse causality.

Mendelian randomization (MR) is a genetic epidemiologic method using genetic variants as instrumental variables to determine the inter-causality between exposure and risk factors related to diseases, avoiding confounding biases inherent in observational studies (20). Because the genotype is randomly assigned during conception, genetic variation is not affected by potential confounding factors (such as environmental exposure), nor is it changed by the occurrence of disease (21). Therefore, the aim of this study was to use a two-step MR analysis to illustrate the causal relationship between EA, brain cortical structure, and sarcopenia based on the available genome-wide association studies (GWASs) public data from a large population, with a view to providing a evidence for the prevention of sarcopenia at the public health level.

## Methods

The study was conducted in accordance with the Declaration of Helsinki (as revised in 2013). The study used publicly available non-identity data from studies of participants in human trials approved by the medical ethics committee. Hence, ethical approval was not needed for our study. Details of ethical approval and written informed consents of participants for each of the studies that contributed to the GWAS can be found in the original publications.

### Data sources

For educational attainment, the definition of educational attainment is the number of years of schooling that participants had completed. Each major educational qualification was mapped to the International Standard Classification of Education to derive the equivalent years of schooling. The GWAS data correlated with years of schooling were obtained from Social Science Genetic Association Consortium (SSGAC) summary data, which was a meta-analysis of 70 cohorts including 766,345 participants of European ancestry (Details of each cohort are shown in Supplementary Table S1) (22).

For brain cortical structure, defined as human brain cortical surface area (SA) and cortical thickness (TH) measured by MRI, the GWAS data correlated with it were obtained from Evidence-based Network for the Interpretation of Germline Mutant Alleles (ENIGMA) Consortium (23, 24). They were the meta results of 60 cohorts around the world including 51,665 participants, primarily (94%) of European descent. Our study used data including only European ancestry (Details of each cohort are shown in Supplementary Table S2).

For sarcopenia, the most widely cited definition nowadays was proposed by the European Working Group on Sarcopenia in Older People (EWGSOP) (25) and updated as EWGSOP2 in January 2019 (26). The diagnosis of sarcopenia requires the measurement of a combination of muscle mass, muscle strength, and physical performance (26). We chose the summary-level GWAS data for three sarcopenia-associated traits, namely appendicular lean mass (ALM), hand grip strength (left and right), and usual walking pace, which were used to assess muscle mass, muscle strength, and physical performance, respectively. The data of ALM were obtained from a GWAS with 450,243 participants from the UK Biobank (27). The GWAS data of hand grip strength (left and right) including 461,026/461,089 participants (left and right) and usual walking pace including 459,915 participants were both obtained from the UK Biobank (28).

### Selection of instrumental variables

In MR analysis, single-nucleotide polymorphisms (SNPs) from the exposure data set are used as instrumental variables. Instrumental variables must satisfy the following three assumptions: (1) The instrumental variables are strongly correlated with the exposure; (2) The instrumental variables are not associated with confounders; (3) The instrumental variables can only affect the outcome through the exposure (29). Instrumental variables for exposure traits were selected according to several criteria: (1): the GWAS-correlated *p*-value <5 × 10^−8^; (2) the linkage disequilibrium [LD] *r*^2^ < 0.001, and < 1 MB from the index variant; (3) F statistics >10 (30).

### Mendelian randomization analysis

This is a two-step MR analysis to illustrate the causal relationship between EA, brain cortical structure, and sarcopenia-associated traits. In this study, brain cortical structure was analyzed as the mediator. The specific causal analysis included the following steps (1) exploring the causal relationship between EA and sarcopenia-associated traits; (2) assessment of the effects of EA on brain cortical structure and brain cortical structure on sarcopenia-associated traits; (3) investigating how brain cortical structure mediates the causal relationship between EA and sarcopenia-associated traits. The random-effect inverse-variance weighted (IVW), MR Egger, and weighted median were used in the MR analyses, in which IVW was used as the main outcome while MR Egger and weighted median were used to complement and validate IVW. For the first step, the Bonferroni-corrected *p*-value was set as 1.25 × 10^−2^ (0.05/4). For the second step, a p-value <0.05 was regarded as significant and the Bonferroni-corrected p-value was set as 6.25 × 10^−3^ (0.05/8). In addition, mediation analysis was conducted on the mediator (brain cortical structure) with potential correlation. The effect of EA on brain cortical structure was multiplied by the effect of brain cortical structure on sarcopenia-associated traits to obtain the mediatory effect of brain cortical structure. The mediatory effect of brain cortical structure was subtracted from the total effect of EA on sarcopenia-associated traits to obtain the direct effect of EA on sarcopenia-associated traits. Moreover, the mediatory effect was divided by the total effect of EA on sarcopenia-associated traits to obtain the proportion of mediation by brain cortical structure.

To further ensure the robustness of our MR analysis, we performed the following sensitivity analyses. The heterogeneity was estimated with the Cochran Q test and significant heterogeneity was indicated if the *p*-value <0.05; the horizontal pleiotropy was assessed using the MR-Egger intercept test and leave-one-out analyses; To further control for potential pleiotropy, we also used the MR Pleiotropy Residual Sum and Outlier (MRPRESSO) to identify potential pleiotropic outlier variables, and repeated random-effect IVW analysis after removing these outlier variables. All analyses were performed using the TwoSampleMR packages in R software (v4.3.0, R Foundation for Statistical Computing, Vienna, Austria).

## Results

For EA, a total of 317 SNPs were selected as the instrumental variables (Supplementary Table S3). For SA (Supplementary Table S4) and TH (Supplementary Table S5), 12 SNPs and 6 SNPs were selected as the instrumental variables, respectively.

### The causal relationship between EA and sarcopenia-associated traits

**Figure 1:**
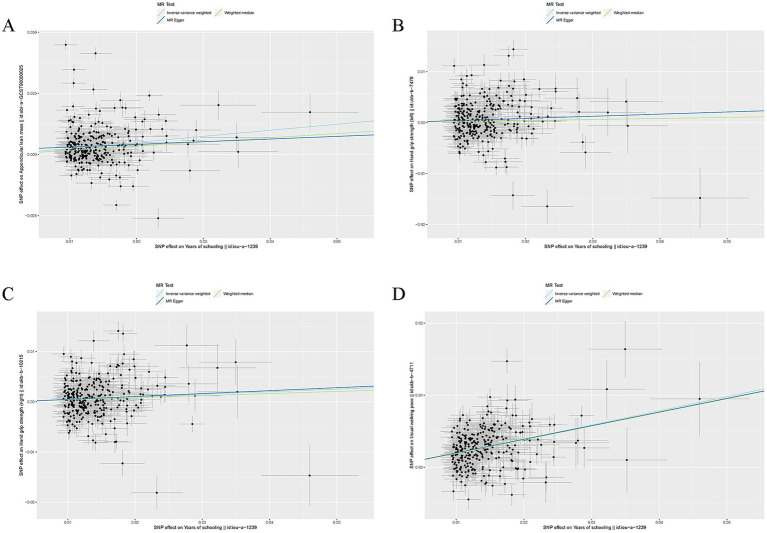
Mendelian randomization analysis between EA and sarcopenia-associated traits. **(A)** Scatter plots from genetically predicted EA on ALM; **(B)** Scatter plots from genetically predicted EA on hand grip strength (left); **(C)** Scatter plots from genetically predicted EA on hand grip strength (right); **(D)** Scatter plots from genetically predicted EA on usual walking pace.

EA was associated with increased ALM (*β* = 0.25, SE = 0.031, *p* = 2.25 × 10^−15^), hand grip strength (left: β = 0.042, SE = 0.015, *p* = 4.77 × 10^−3^ and right: β = 0.050, SE = 0.015, *p* = 5.17 × 10^−4^), and usual walking pace (β = 0.20, SE = 0.010, *p* = 6.16 × 10^−83^) (Table 1). Other details are presented in Figure 1.

**Table 1:** Mendelian randomization analysis between EA, brain cortical structure, and sarcopenia-associated traits.

Exposure	Outcome	IVW-derived *p* value	β (95% confidence intervals)	Cochran Q-derived *P* value	MR-Egger intercept derived *p* value
EA	ALM	2.25 × 10^−15^	0.25 (0.19 to 0.31)	0	0.24
	Hand grip strength (left)	4.77 × 10^−3^	0.042 (0.013 to 0.071)	9.38 × 10^−127^	0.9994
	Hand grip strength (right)	5.17 × 10^−4^	0.050 (0.022 to 0.079)	1.68 × 10^−117^	0.88
	Usual walking pace	6.16 × 10^−83^	0.20 (0.18 to 0.22)	2.78 × 10^−53^	0.86
	SA	3.40 × 10^−7^	4082.36 (2513.35 to 5681.38)	1.99 × 10^−30^	0.58
	TH	3.45 × 10^−3^	0.014 (0.0045 to 0.023)	1.61 × 10^−9^	0.28
SA	ALM	0.10	9.28 × 10^−6^ (−1.89 × 10^−6^ to 2.04 × 10^−5^)	3.25 × 10^−175^	0.59
	Hand grip strength (left)	0.012	3.79 × 10^−6^ (8.16 × 10^−7^ to 6.75 × 10^−6^)	5.43 × 10^−6^	0.044
	Hand grip strength (right)	0.017	3.81 × 10^−6^ (6.95 × 10^−7^ to 6.93 × 10^−6^)	7.04 × 10^−18^	0.11
	Usual walking pace	0.081	1.98 × 10^−6^ (−2.42 × 10^−7^ to 4.20 × 10^−6^)	1.37 × 10^−11^	0.12
TH	ALM	0.83	0.10 (−0.86 to 1.1)	6.63 × 10^−9^	0.42
	Hand grip strength (left)	0.31	−0.46 (−1.34 to 0.42)	2.36 × 10^−12^	0.49
	Hand grip strength (right)	0.37	−0.40 (−1.27 to 0.48)	4.42 × 10^−12^	0.55
	Usual walking pace	2.69 × 10^−3^	0.49 (0.17 to 0.81)	0.041	0.94

Educational attainment, EA; Inverse variance weighted, IVW; Appendicular lean mass, ALM; Cortical surface area, SA; Cortical thickness, TH.

For all four MR estimates, *p* values of the Cochran Q tests were < 0.05 and showed heterogeneity. As we used the random-effects IVW as the main result, heterogeneity is acceptable (31). The MR-Egger intercept tests did not show evidence of directional pleiotropy in any of the analyses, and there was no distortion in any of the leave-one-out plots (Supplementary Figure S1). MRPRESSO identified no outliers.

### The effects of EA on brain cortical structure

**Figure 2:**
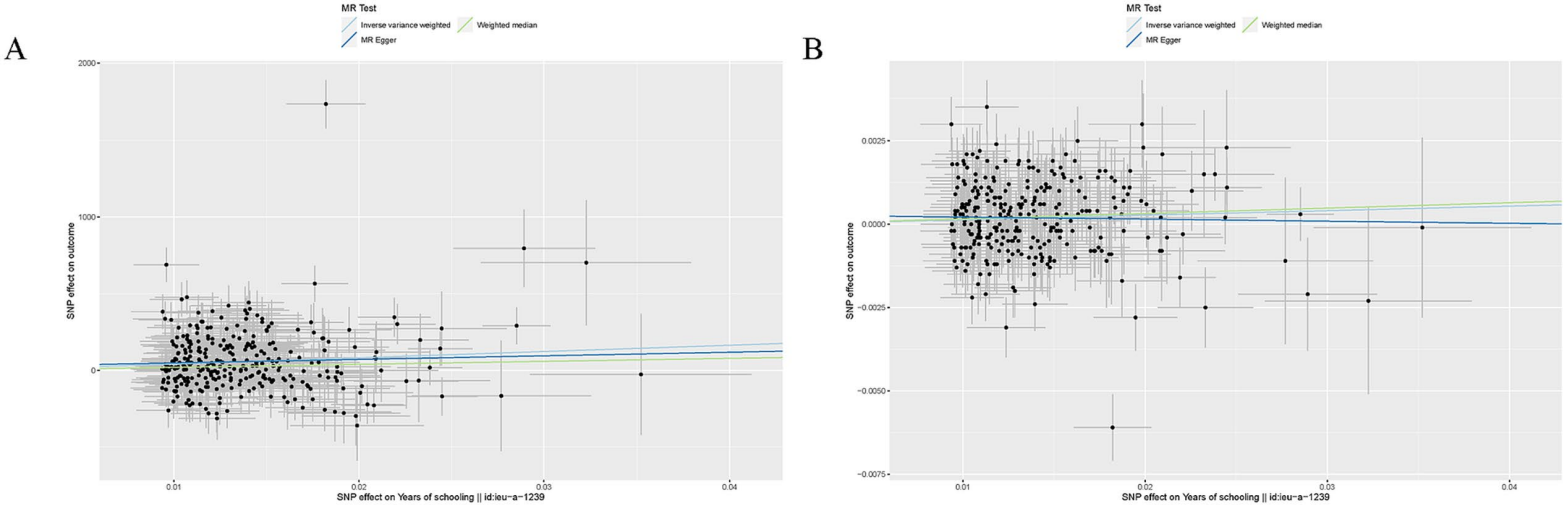
Mendelian randomization analysis between EA and brain cortical structure. **(A)** Scatter plots from genetically predicted EA on SA; **(B)**. Scatter plots from genetically predicted EA on TH.

EA was associated with increased SA (*β* = 4082.36, SE = 800.52, *p* = 3.40 × 10^−7^) and TH (β = 0.014, SE = 0.0046, *p* = 3.45 × 10^−3^) (Table 1). Other details are presented in Figure 2.

For both two MR analyses, Cochran Q-derived *p* values were also <0.05 and showed heterogeneity. No directional pleiotropy was found in the MR-Egger intercept tests. No outlier between EA on brain cortical structure was identified by MRPRESSO, and the robustness of results was confirmed by the leave-one-out plots (Supplementary Figure S2).

### The effects of brain cortical structure on sarcopenia-associated traits

For SA and TH, only TH was found to increase the usual walking pace (*β* = 0.49, SE = 0.16, *p* = 2.69 × 10^−3^). The causal effects of SA on sarcopenia-associated traits and TH on ALM and hand grip strength (left and right) were not established (Table 1). Other details are presented in Supplementary Figure S3.

Cochran Q-derived *p* values were < 0.05 and showed heterogeneity. No directional pleiotropy was found in the MR-Egger intercept tests and MRPRESSO identified no outliers. There was no distortion in the leave-one-out plots (Supplementary Figure S4).

## Mediation analysis

Because only TH has a causal relationship with sarcopenia-associated traits, only TH was analyzed for mediation. Regarding the causal effect of EA on usual walking pace, the mediatory effect of TH was 0.0069 and the proportion of mediation by TH was 3.43%. In addition, the direct effect of EA on the usual walking pace was 0.19.

## Discussion

To our knowledge, this was the first MR analysis using large-scale GWAS data that revealed the causal relationship between EA, brain cortical structure, and sarcopenia. In our study, we revealed strong evidence in support of a protective causal effect of EA on sarcopenia-associated traits including ALM, hand grip strength (left and right), and usual walking pace. Moreover, we also found EA could affect the brain cortical structure including SA and TH, and the brain cortical structure could mediate the protective effect of EA against sarcopenia risk, mainly through the effect of TH on the usual walking pace. It also provided evidence for the existence of muscle-brain cross-talk.

Sarcopenia is an age-related disease with high incidence and serious consequences in the older adult and has become a major public health issue, especially in the context of an aging population (1). As the pathogenesis of the disease is not clear, it brings difficulties to the treatment and prevention of sarcopenia. The studies found that the incidence of sarcopenia was very high in people with lower socioeconomic status (9, 10, 32). In addition, it was found that higher level EA was associated with a lower incidence of sarcopenia (8–12). Then, as a major indicator of socioeconomic status, EA is expected to become a potential intervention factor to solve the problem of high incidence in people with low socioeconomic status. In our study, we confirmed the protective causal effect of EA on sarcopenia-associated traits while minimizing biases due to confounding and reverse causation. This will provide important inspiration for the prevention of sarcopenia. Findings from a study revealed that directly intervening on EA by raising the school-leaving age has been shown to be effective in improving health including reduced risks of diabetes and reduced mortality (33). Although in our study, we defined EA by the number of years of schooling, it is obvious that it is not the only indicator that affects EA. For example, educational opportunities and educational quality also need to be considered. All in all, our results show that education policy reforms at the population level are effective for the prevention of sarcopenia. We hope that the education department and the public health department can collaborate effectively and formulate reasonable policy reforms to effectively prevent sarcopenia, a public health problem in aging.

After confirming the causal relationship between EA and sarcopenia, we explored the possible mechanism by which EA reduces the risk of sarcopenia. The results showed that EA could affect the brain cortical structure including SA and TH, and the brain cortical structure could mediate the protective effect of EA against sarcopenia risk, mainly through the effect of TH on the usual walking pace. The founding that EA could affect the brain cortical structure was consistent with the results of the existing observational neuroimaging literature (16, 17, 23), and we have confirmed this causal relationship again by the genetic epidemiologic method. The mechanism of EA affecting the structure of the cerebral cortex may come from two aspects, one is to promote cranial nerve development in children or adolescents, and the other is to protect and slow down cranial nerve degeneration in adulthood or old age (34). At present, some studies have also tried to distinguish the effects of EA on neurodevelopment and neurodegeneration. One study addressed this problem by exploring the relationship between EA and total gray matter volume and intracranial volume in a cohort covering childhood and older age (4–97 years) (35). This study found that there was a consistent positive relationship between EA and total gray matter volume and intracranial volume in early childhood development, but there was no evidence that EA was related to the protective effect of total gray matter volume and intracranial volume in old age, indicating that the effect of EA on cranial nerve is mainly the promotion of development in childhood, rather than the protective effect of preventing neurodegeneration in old age. This conclusion has also been confirmed by several studies (36, 37). The mediating effect of the brain cortical structure on the prevention of sarcopenia by EA may be realized by the confirmed muscle-brain cross-talk (19). The brain affects the muscles through the regulation of neuromuscular junctions, and in turn, the muscles affect the brain through a series of cytokines such as myokine. Study by Lu et al. added evidence for muscle-brain cross-talk (38). Our results further confirmed the existence of the muscle-brain axis.

Although this study can draw more reliable conclusions by using large-scale genetic variation data, some limitations of the study still need to be considered. First of all, all GWAS data come from the European population, and it is not clear whether our conclusions can be extended to other populations. Second, although we have used data measured by MRI to evaluate the brain cortical structure, we cannot determine what extent these data capture pre-morbid brain cortical structure data, rather than neuropathological changes secondary to the disease. Third, we only studied the overall change data of the brain cortical structure, but not the data of the specific structure of the cerebral cortex. Finally, sample overlap between GWAS studies may have biased MR estimates toward observational association estimates.

## Conclusion

The study will have revealed the protective causal effect of EA on sarcopenia, which provides a reference for the prevention of sarcopenia at the public health level. We also will have found EA could affect the brain cortical structure, and the brain cortical structure could mediate the protective effect of EA against sarcopenia risk.

## Data Availability

The original contributions presented in the study are included in the article/Supplementary material, further inquiries can be directed to the corresponding author.
