# Supplementary material for: Educational attainment, brain cortical structure, and sarcopenia: a Mendelian randomization study
Source: Front Public Health. 2024 Oct 23;12:1415343. doi: 10.3389/fpubh.2024.1415343 (PMC11538070; doi:10.3389/fpubh.2024.1415343)
Supplement: Supplementary file 2 [file Data_Sheet_2.docx]

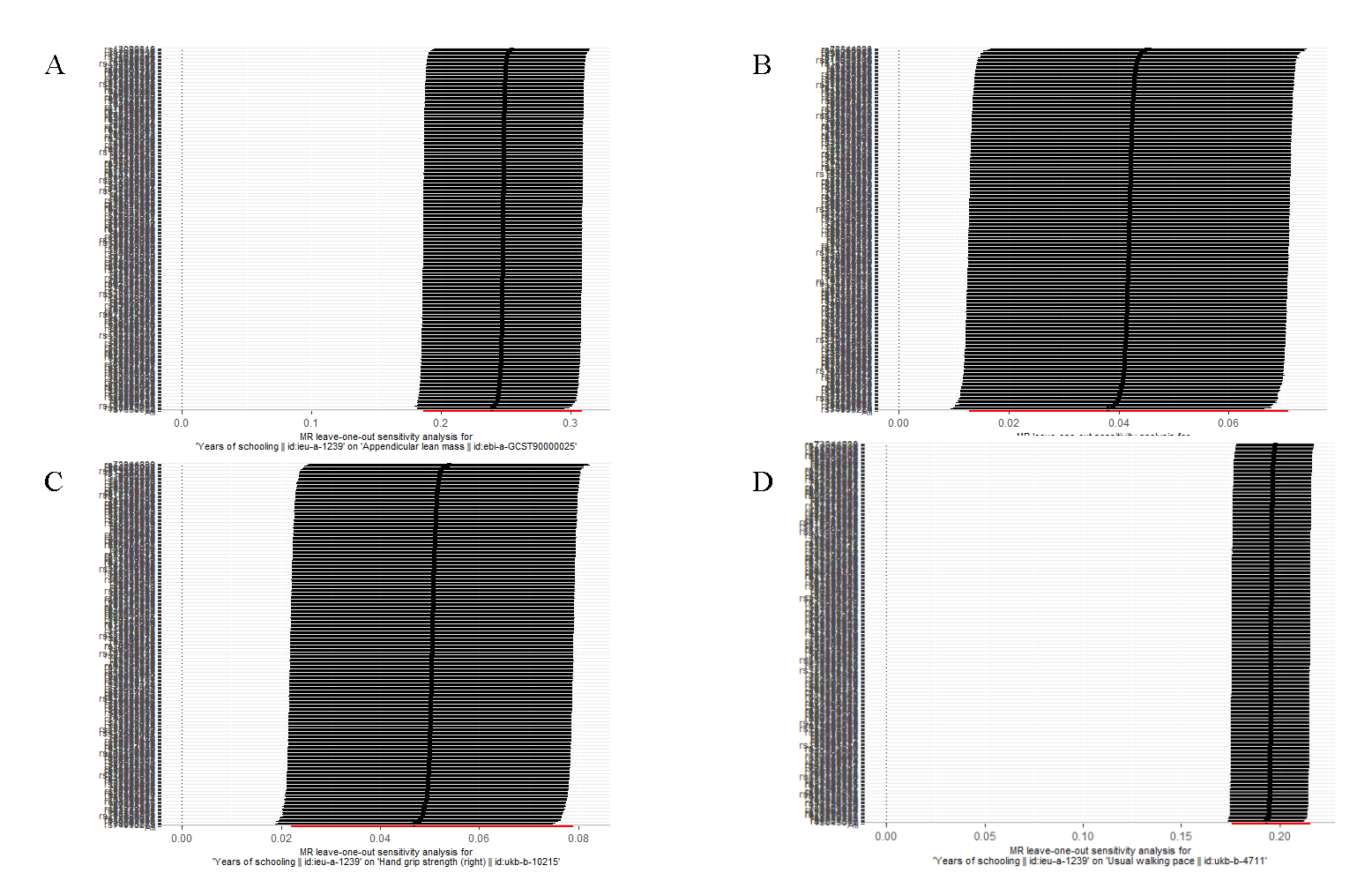


**Figure S1.** Leave-one-out plots from genetically predicted EA on **A.** ALM; **B.** hand grip strength (left); **C.** hand grip strength (right); **D.** usual walking pace.


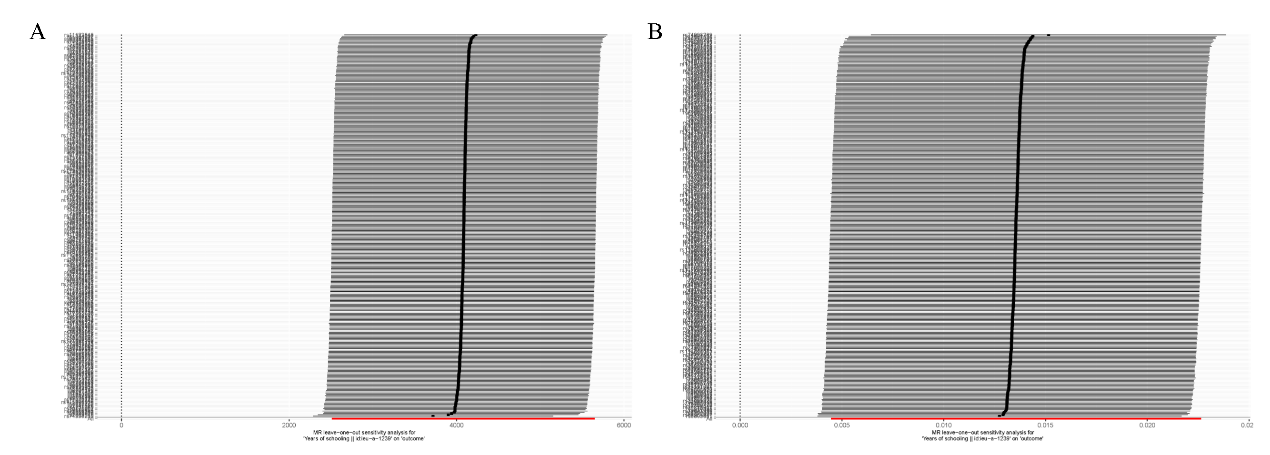


**Figure S2.** Leave-one-out plots from genetically predicted EA on **A.** SA; **B.** TH.


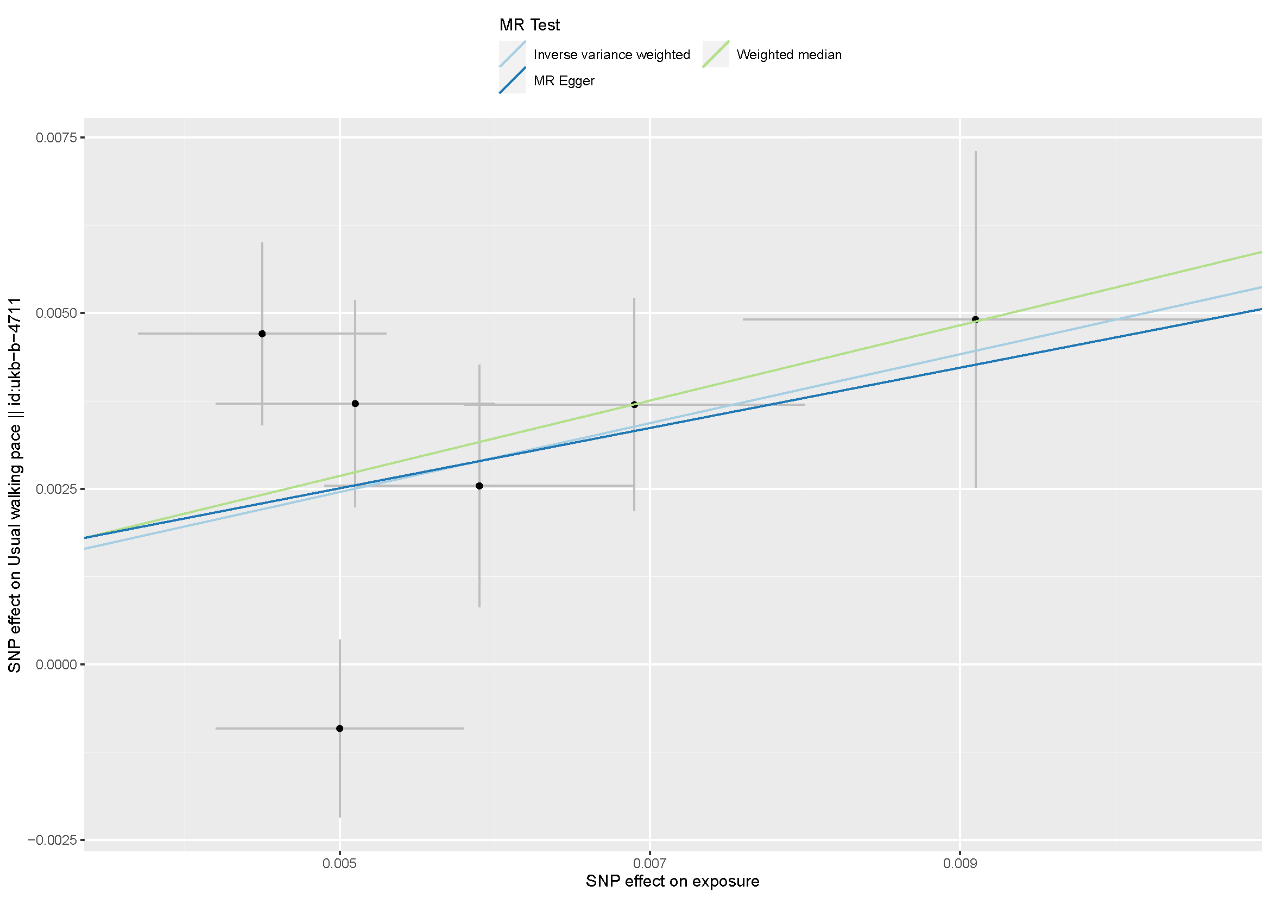


**Figure S3.** Mendelian randomization analysis between TH and usual walking pace.


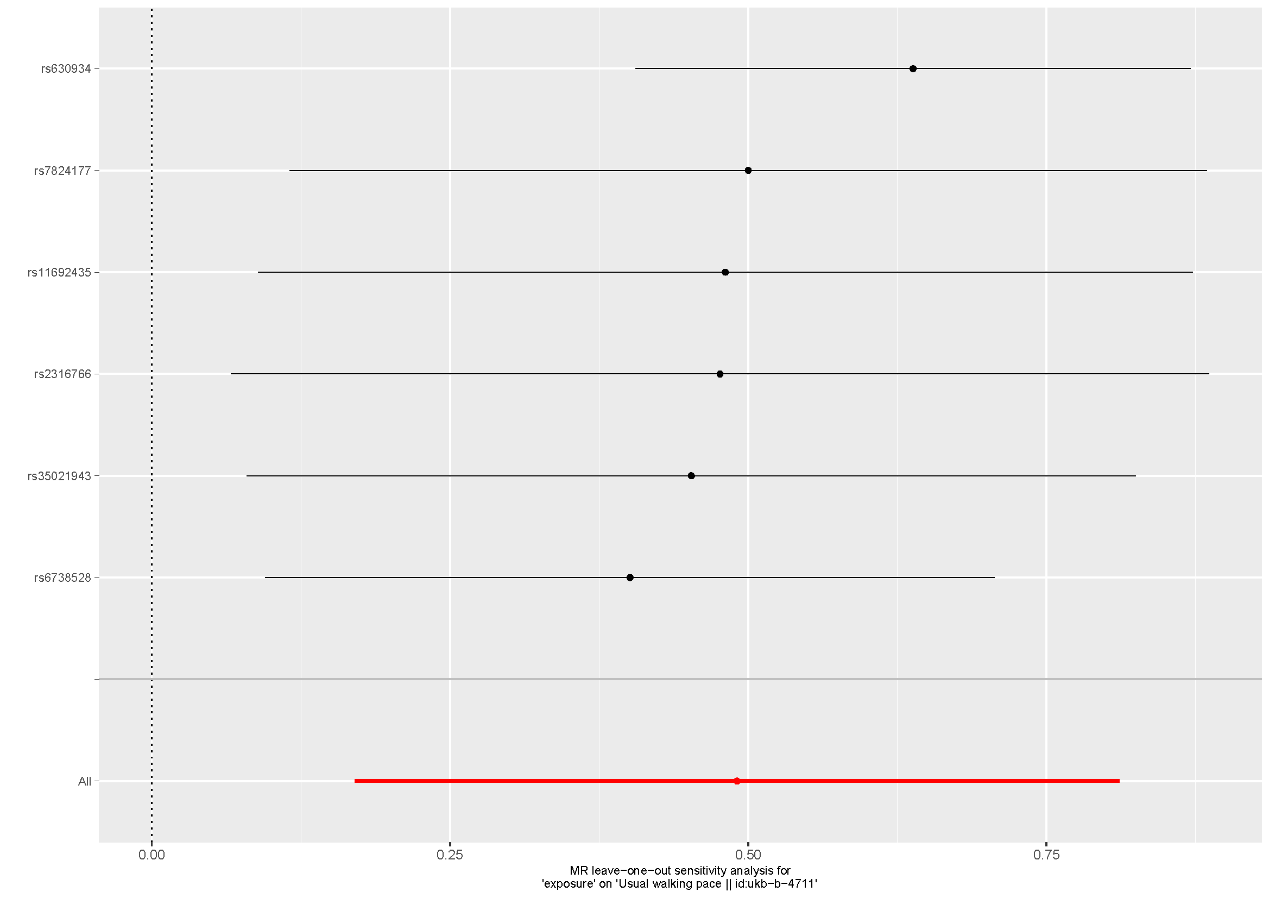


**Figure S4.** Leave-one-out plots from genetically predicted TH on usual walking pace
